# Implant failure and postoperative complications after stabilization surgery for spinal metastases: A single-center cohort study

**DOI:** 10.1016/j.bas.2026.106088

**Published:** 2026-05-09

**Authors:** Santhosh G. Thavarajasingam, Ahmed Salih, Christine Brühl, Daniel Scurtu, Sree Kanakala, Srikar Namireddy, Daniele Ramsay, Ahkash Thavarajasingam, Tim-Mathis Beutel, Jonathan Neuhoff, Josephine Pollok, Merih Turgut, Dragan Jankovic, Florian Ringel, Andreas Kramer

**Affiliations:** a Department of Neurosurgery, LMU University Hospital, LMU Munich, Germany; b Imperial Brain & Spine Initiative, Imperial College London, United Kingdom; c Department of Neurosurgery, University Medical Center Mainz, Germany; d Faculty of Medicine, Imperial College London, United Kingdom; e Center for Spinal Surgery and Neurotraumatology, Berufsgenossenschaftliche Unfallklinik Frankfurt am Main, Frankfurt, Germany

**Keywords:** Spinal metastases, Stabilization surgery, Postoperative infection, Implant failure, Complications

## Abstract

**Study design:**

Retrospective single-center cohort study.

**Objective:**

To evaluate associations between clinical, radiographic, and surgical factors and the risk of implant failure and postoperative complications following spinal stabilization for metastatic disease.

**Summary of background data:**

Spinal metastases frequently cause pain, neurological deficits, and instability, often requiring surgical decompression and stabilization. As survival improves and the use of spinal instrumentation increases, understanding surgery-associated complication risks has become increasingly important. While postoperative complications in metastatic spine surgery have been reported across multiple cohorts, factors related to construct design and anchorage, including implant failure, wound infection, and postoperative bleeding, remain incompletely characterized. This study examines the incidence of these complications and their associated factors in patients undergoing stabilization for metastatic spinal disease.

**Methods:**

This retrospective study retrospectively analyzed 149 patients who underwent stabilization surgery for spinal metastases between 2010 and 2020. Instability was assessed using the Spinal Instability Neoplastic Score (SINS), functional status using the Karnofsky Performance Score, and neurological status using the Frankel classification. Associations between patient-, disease-, and surgery-related variables and postoperative outcomes were examined primarily using univariate logistic regression analyses.

**Results:**

In univariate analyses, implant failure was significantly associated with instrumentation anchored in metastatically involved adjacent vertebrae (OR 9.22), reoperation for tumor progression (OR 8.25), preoperative Frankel Score E (OR 5.25), active smoking (OR 4.05), and postoperative wound infection (OR 4.88). Postoperative wound infection was associated with construct anchorage in metastatically involved adjacent vertebrae (OR 4.78) and a preoperative ACE-27 comorbidity score of 1 (OR 3.54). Postoperative bleeding or hematoma was associated with wound infection (OR 5.21), advanced age at surgery (OR 1.09), impaired preoperative mobility (OR 3.78), and lower postoperative Karnofsky Performance Score (OR 0.96). The Spinal Instability Neoplastic Score was associated with implant failure in univariate analysis but did not account for construct-end anchorage.

**Conclusions:**

Anchoring spinal constructs in vertebrae affected by metastases was consistently associated with increased risks of implant failure and postoperative infection following stabilization surgery. These findings highlight the mechanical and biological vulnerability of metastatic bone and suggest that, when feasible, fixation into structurally uninvolved vertebrae may reduce postoperative complications.

## Highlights

• End anchorage in tumor-affected vertebrae raised failure 9-fold.
• Wound infection and implant failure each raised the other's odds 5-fold.
• While SINS remains valuable for assessing baseline instability, it did not retain an association with implant failure after adjustment, suggesting that radiographic instability alone may be insufficient to capture the mechanical challenges of metastatic fixation.
• Extending construct ends into healthy bone may lower complication risk.

## Teaser

This retrospective cohort study evaluated associations between clinical, radiographic, and surgical factors and postoperative complications following stabilization surgery for spinal metastases in 149 patients. Anchoring fixation constructs in metastatically involved vertebrae was consistently associated with higher risks of implant failure and wound infection. Tumor progression, active smoking, and functional status were also associated with implant failure. Postoperative bleeding was associated with wound infection, older age, and lower Karnofsky Performance Score, highlighting the multifactorial nature of complications in this population.

## 1 Key points

• Anchoring screws in metastatically involved vertebrae was strongly associated with increased risks of implant failure and wound infection.
• Implant failure was associated with tumor progression, active smoking, and preoperative Frankel E status after adjustment.
• Postoperative bleeding was associated with wound infection, older age, and lower preoperative Karnofsky Performance Score.
• The Spinal Instability Neoplastic Score (SINS) was associated with implant failure on univariate analysis, but not after multivariable adjustment.
• Complication risk following metastatic spinal fixation appears to reflect interacting mechanical, biological, and patient-related factors rather than radiographic instability alone.

## 2 Introduction

Spinal metastases represent the most common malignant bone tumors and affect approximately 15% of all patients with cancer, with peak incidence between 40 and 65 years of age. Autopsy studies suggest that these metastases are often underdiagnosed, with higher true prevalence rates (Van den et al., 2022; Wright et al., 2018; Li et al., 2021). They frequently lead to pain, neurological deficits, and structural instability, significantly impairing patients’ quality of life. As advances in oncological care prolong survival, therapeutic strategies must be continuously reassessed to balance disease control with functional outcomes. Several prognostic scoring systems have been developed to estimate survival and guide treatment decisions (Schoenfeld et al., 2021; Balain et al., 2013; Karhade et al., 2019, 2020; Kramer et al., 2022), and the neurologic, oncologic, mechanical, and systemic (NOMS) decision framework has become central to therapy selection in spinal metastases (Laufer et al., 2013).

A landmark study by Patchell et al., in 2005 demonstrated that surgical decompression combined with radiation therapy significantly benefits patients with neurological deficits from epidural spinal cord compression, compared to radiation alone (Patchell et al., 2005). Subsequent advances in radio-oncology, such as high-dose stereotactic irradiation, further emphasized the need for surgical separation of metastases from the spinal cord to prevent radiation-induced injury (Bate et al., 2015). This led to the development of *separation surgery*, combining decompression and stabilization to protect neural structures and restore mechanical integrity.

Beyond decompression in cases of myelopathy or instability, load-bearing instrumentation is increasingly used to alleviate pain and improve quality of life. In particular, instrumentation plays a central role in the treatment of pathological fractures and impending mechanical collapse caused by metastatic vertebral destruction. As oncological care improves survival, the durability of these constructs and the complications associated with them become increasingly relevant to surgical decision-making. As surgical indications broaden and instrumentation has become more common in the treatment of spinal metastases, understanding the associated complication risks becomes critical. While postoperative complication rates following surgery for spinal metastases have been reported in multiple retrospective and prospective cohorts, fewer studies have specifically examined construct-end anchorage in vertebrae affected by metastases as a focused mechanical factor associated with implant failure and wound complications.

Accordingly, the present study evaluates postoperative complications following stabilization surgery for spinal metastases in a single-center cohort. We specifically examine associations between patient-, disease-, and surgery-related factors and the risk of implant failure, wound infection, and postoperative bleeding. We hypothesized that construct-related variables - particularly anchoring instrumentation in metastatically involved vertebrae - would be associated with an increased risk of postoperative complications.

## 3 Methods

### 3.1 Patient selection and data collection

This retrospective single-center cohort study was conducted by screening the institutional departmental database to identify all consecutive patients who underwent surgical stabilization, with or without decompression of neural structures, for spinal metastases between January 2010 and December 2020. Patients were included if stabilization surgery was performed for metastatic involvement of the spine, irrespective of primary tumor type, spinal level, or surgical approach. No additional exclusion criteria were applied.

### 3.2 Clinical and surgical variables

Comprehensive demographic, clinical, radiographic, and surgical data were extracted from electronic medical records. Preoperative spinal stability was assessed using the Spinal Instability Neoplastic Score (SINS), which was independently calculated by two investigators for each case. Functional status was evaluated using the Karnofsky Performance Score (KPS), comorbidity burden using the Adult Comorbidity Evaluation-27 (ACE-27), and neurological function using the Frankel classification system.

Surgical variables included approach, instrumentation strategy, and extent of fixation. More granular implant-specific variables, including cement augmentation, exact construct length, junctional segment involvement, and minimally invasive versus open instrumentation technique, were not consistently documented across the study period and were therefore not included in the formal analysis. Implant failure was defined as mechanical failure of the instrumentation requiring revision surgery. Additional postoperative complications assessed included wound infection requiring surgical revision, postoperative bleeding or hematoma, new neurological deficits, implant misplacement, and cerebrospinal fluid leakage. Particular attention was given to cases in which the cranial or caudal end of the construct was anchored in vertebrae affected by metastatic disease. For clarity, this variable was analyzed as construct anchorage in metastatically involved adjacent vertebrae. Mortality data were obtained through linkage with the institutional cancer registry. Preoperative Frankel grade was analyzed as a binary variable (Frankel E versus Frankel A–D to distinguish neurologically intact from neurologically impaired patients.

### 3.3 Definition of metastatic involvement at construct ends

For the purpose of this study, metastatic involvement at the cranial or caudal anchoring level was defined in a binary manner. A vertebra was considered metastatically involved if preoperative CT and/or MRI demonstrated tumor infiltration of the vertebral body and/or posterior elements, as documented in the formal radiology report. No further stratification was performed with respect to lesion morphology (lytic, blastic, or mixed), degree of vertebral destruction, or tumor volume. Accordingly, this variable captures the presence of metastatic involvement rather than the severity of structural compromise.

### 3.4 Statistical analyses

All statistical analyses were performed using R (R Foundation for Statistical Computing, version 4.1.2). Continuous variables are presented as means with standard deviations or medians with interquartile ranges, as appropriate, and categorical variables as counts and percentages. Group comparisons were performed using chi-square or Fisher's exact tests for categorical variables and Student's *t*-tests or Wilcoxon rank-sum tests for continuous variables, based on distributional assumptions.

Given the exploratory nature of this retrospective cohort and the limited number of outcome events, univariate logistic regression analyses were predefined as the primary analytical approach to evaluate associations between clinical, radiographic, and surgical variables and postoperative outcomes (implant failure, wound infection, and postoperative bleeding).

A broad set of clinically plausible variables was prespecified for univariate screening based on routine clinical practice and prior literature. Given low event counts, some univariate models exhibited complete or quasi-complete separation, resulting in non-estimable confidence intervals (reported as NA).

Odds ratios (ORs) with 95% confidence intervals (CIs) were calculated for each variable individually. Clinically plausible variables that did not reach statistical significance were retained and reported to ensure transparency.

Secondary multivariable logistic regression analyses were performed in an exploratory manner only, to provide adjusted context for selected associations while acknowledging limited statistical power. Candidate variables were selected *a priori* based on clinical relevance and prior literature rather than statistical significance alone. Model refinement was performed using bidirectional stepwise selection based on Akaike's Information Criterion (AIC).

Owing to low event counts for certain outcomes, multivariable models were not intended for prediction or causal inference and were interpreted cautiously as adjusted association analyses. No interaction testing or formal model validation was performed. Statistical significance was defined as a two-sided *p* value ≤ 0.05.

### Ethical approval

This study was approved by the local ethical committee (research no. 2021-16187), and all surgical procedures were conducted in accordance with Good Clinical Practice Guidelines. Informed consent was obtained from all subjects or their legal guardians.

## 4 Results

### 4.1 Patient demographics and disease characteristics

A summary of patient and disease characteristics is depicted in Supplemental Table S1. This study involved 149 patients, predominantly male (65.1%), undergoing stabilization surgery for spinal metastases. The average age at surgery was 65 years, ranging from 28 to 91 years. Among them, 53.7% were non-smokers, 25.5% were active smokers, and 20.8% were former smokers at the time of the operation. A diverse array of primary tumor entities was observed, with lung cancer (24.2%) being the most common, followed by prostate (17.4%) and breast cancers (14.1%). Lesser common origins included renal (8.7%) and hepatic (5.4%), with colorectal and esophageal cancers each at 4.7 % and 4.0%, respectively, and thyroid cancer also at 4.0%. Urothelial tumors and "Cancer of Unknown Primary" or squamous cell carcinoma were noted in smaller percentages.

### 4.2 Preoperative Spinal Instability Neoplastic Score (SINS)

The mean SINS was 10: Affected spinal segments of 69.8% (104 patients) were rated potentially unstable, of 20.8% (31 patients) as unstable, and of 8.7% (13 patients) as stable. The bone lesion types were predominantly lytic (72,5 %), indicative of significant bone loss and fracture risk, while 11.4% had osteoblastic lesions, and 15.4% exhibited mixed characteristics.

### 4.3 Surgical approaches and sites of operation

The majority of surgeries were performed using a dorsal approach (71.8 %), with fewer patients undergoing ventral (10.1%) or ventrodorsal 360° approaches (18.1 %). The thoracic spine was the most frequently operated anatomical spinal region.

### 4.4 Functional & neurological outcomes

The Karnofsky Performance Score (KPS) had a median and median average deviation (MAD) of 60% ± 10 preoperatively. Postoperatively, this improved to an average of 70% ± 10. Immobilizing axial pain, which affected 78.5 % of patients before surgery, was reduced to 45.0% after surgical intervention. Neurological status, assessed using the Frankel Score, demonstrated significant improvement post-surgery. Preoperatively, 42.3 % of patients had no neurological deficits (Frankel Score E), increasing to 51.7% postoperatively. This represents 13 patients (8.7%) who achieved complete neurological recovery post-surgery, as they had deficits preoperatively but recovered fully. In total, 20 patients (13.4%) exhibited partial neurological improvement Despite these improvements, a minority of patients continued to experience significant neurological deficits postoperatively, highlighting the complexity of managing spinal metastases in this population.

### 4.5 Postoperative surgical complications

Surgical complications remain a significant consideration in stabilization surgery for spinal metastases. Short term postoperative mortality was observed in 2 patients (1.3%), while overall mortality reached 77.2 % (115 patients) during the study period.

Wound healing disorders, including wound infections requiring revision surgery, affected 16 patients (10.7%). Postoperative hematoma, including epidural bleeds, occurred in 12 patients (8.1 %). Implant failure necessitating reoperation occurred in 9 patients (6.0%).

## 5 Associations with implant failure

Implant failure requiring revision occurred in 9 patients (6.0%).

**Fig. 1:**
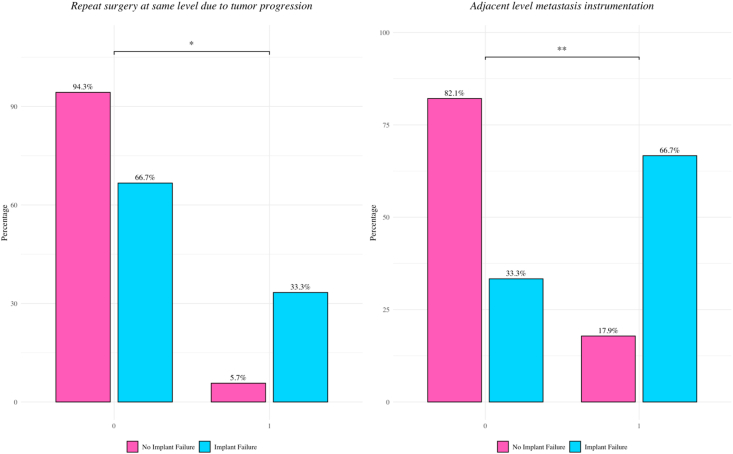
**Patient and disease characteristics significantly associated with implant failure (p < 0.05).** Bar plots showing significant associations between selected clinical and surgical variables and the occurrence of implant failure (p < 0.05, chi-square test). Pink bars indicate patients without implant failure, and blue bars indicate patients with implant failure; percentages are displayed for each group. In the left panel, implant failure occurred more frequently in patients who underwent repeat surgery at the same spinal level due to tumor progression (33.3%) compared with those without repeat surgery (5.7%) (p < 0.05). In the right panel, implant failure was more common when the cranial or caudal end of the construct was anchored in metastatically involved adjacent vertebrae (66.7%) compared with constructs anchored in uninvolved vertebrae (17.9%) (p < 0.01).

On χ^2^ analysis, implant failure was more frequent in cases where the cranial or caudal end of the construct was anchored in metastatically involved vertebrae (66.7% vs. 17.9%, *p* = 0.0013; Fig. 1). Reoperation due to tumor progression was also associated with higher rates of implant failure (33.3% vs. 5.7%, *p* = 0.0158).

Univariate logistic regression (Table 2) demonstrated significantly increased odds of implant failure associated with construct anchorage in metastatically involved adjacent vertebrae (OR 9.22, *p* = 0.0027), tumor progression requiring reoperation (OR 8.25, *p* = 0.0080), preoperative Frankel Score E (OR 5.25, *p* = 0.043), active smoking (OR 4.05, *p* = 0.0455), and postoperative wound infection (OR 4.88, *p* = 0.038). Baseline neurological deficits were associated with lower odds of implant failure (OR 0.20, *p* = 0.047).

**Table 1:** Univariate regression analysis results for implant failure (p < 0.05).

Variable	Coefficient	Odds Ratio (OR)	CI: Lower	CI: Upper	*p*-Value
*Adjacent level metastasis instrumentation*	2.22	9.22	2.27	45.97	0.0027
*Reoperation at the same spinal level due to tumor progression*	2.11	8.25	1.53	38.19	0.0080
*Active smoker*	1.4	4.05	1.02	17.21	0.0455
*Postoperative wound infection*	1.58	4.88	0.95	20.98	0.038
*Preoperative Frankel Score: E*	1.66	5.25	1.22	36.12	0.043
*Neurological symptoms preoperative*	−1.63	0.20	0.03	0.85	0.047

Table 1 presents the results of the univariate logistic regression analyses examining associations with implant failure following spinal stabilization surgery for metastatic disease. Adjacent level metastasis instrumentation was strongly associated with implant failure (OR = 9.22, 95% CI: 2.27–45.97, p = 0.0027). Reoperation at the same spinal level due to tumor progression was also associated with an increased risk of implant failure (OR = 8.25, 95% CI: 1.53–38.19, p = 0.0080). Additional variables associated with implant failure included active smoking (OR = 4.05, 95% CI: 1.02–17.21, p = 0.0455), postoperative wound infection (OR = 4.88, 95% CI: 0.95–20.98, p = 0.038), and preoperative Frankel Score E (OR = 5.25, 95% CI: 1.22–36.12, p = 0.043). In contrast, the presence of neurological symptoms before surgery was associated with lower odds of implant failure (OR = 0.20, 95% CI: 0.03–0.85, p = 0.047). Variables evaluated but not significantly associated with implant failure included demographic factors (age at diagnosis, age at surgery, sex, body mass index, height, weight), survival status, tumor characteristics (primary tumor type), metastatic burden (synchronous or metachronous bone metastases; visceral, pulmonary, hepatic, cerebral, adrenal, lymph node, soft tissue, and other distant metastases), number and anatomical distribution of vertebral body metastases, affected spinal regions (cervical, thoracic, lumbar, sacral), pre- and postoperative Karnofsky Performance Score, overall and individual ACE-27 comorbidities, former smoking status, prior spinal surgery (same or different level), spinal instability before or after surgery, preoperative Frankel grades A–D, preoperative mobility level, presence of ataxia, surgical approach and technique, staged procedures, stabilization-only surgery, postoperative pain response, implant misplacement, and postoperative hematoma or bleeding.

**Table 2:** Univariate regression model for post-operative wound infection (p < 0.05).

*Variable*	Coefficient	Odds Ratio	CI: Lower	CI: Upper	*p-*Value
*Adjacent level metastasis instrumentation*	1.57	4.78	1.60	14.32	0.0044
*Preoperative ACE 27 Score: 1*	1.26	3.54	1.17	13.18	0.0360
*Implant failure*	1.59	4.88	0.95	20.99	0.0380

Table 2 presents the results of the univariate logistic regression analyses examining associations with postoperative wound infection following stabilization surgery for spinal metastases. Adjacent level metastasis instrumentation was associated with an increased risk of postoperative wound infection (OR = 4.78, 95% CI: 1.60–14.32, p = 0.0044). A preoperative ACE-27 comorbidity score of 1 was also associated with higher odds of wound infection (OR = 3.54, 95% CI: 1.17–13.18, p = 0.0360). In addition, implant failure was associated with an increased risk of postoperative wound infection (OR = 4.88, 95% CI: 0.95–20.99, p = 0.0380). Variables evaluated but not significantly associated with postoperative wound infection included demographic factors (age at surgery, sex, body mass index), smoking history (current or former), primary tumor type, metastatic burden (visceral and distant metastases), number and anatomical distribution of vertebral metastases, affected spinal regions (cervical, thoracic, lumbar, sacral), preoperative and postoperative functional and neurological status (Karnofsky Performance Score, ECOG status, Frankel grade), presence of ataxia, surgical indication, surgical approach and technique (spondylodesis, laminectomy, kyphoplasty/vertebroplasty, corpectomy; dorsal, ventral, or combined approaches), postoperative pain response, implant misplacement, and other surgery-related complications including dural leak and postoperative hematoma or bleeding.

Exploratory multivariable logistic regression was performed to assess adjusted associations; results are shown in Supplemental Table S2 and Fig. 4 and are interpreted cautiously given the limited number of failure events.

## 6 Associations with wound infection

Postoperative wound infection requiring revision occurred in 16 patients (10.7%).

**Fig. 2:**
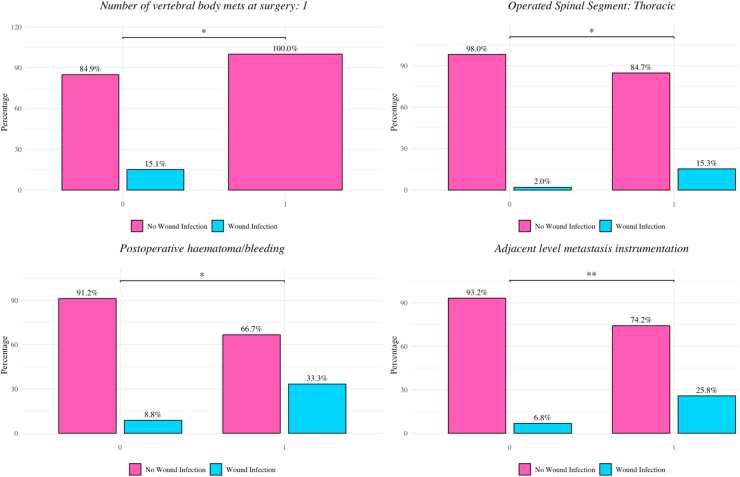
**Patient and disease characteristics significantly associated with postoperative wound infections (p < 0.05).** Bar plots showing significant associations between clinical and procedural variables and the occurrence of post-operative wound infections (p < 0.05, Chi-square test). Pink bars indicate patients without wound infection, blue bars indicate those with infection, with percentages displayed for each group. In the top left panel, patients with only one vertebral body metastasis at surgery showed a significantly higher infection rate (15.1%) compared to those without (0%) (*p* < 0.05). The top right panel highlights that thoracic spinal segment operations were associated with a higher infection risk (15.3%) versus 2.0% in patients without thoracic involvement (*p* < 0.05). In the bottom left panel, postoperative hematoma or bleeding was significantly associated with wound infections, occurring in 33.3% of cases with bleeding compared to 8.8% without (*p* < 0.05). The bottom right panel shows that adjacent level metastasis with instrumentation significantly increased wound infection risk (25.8% vs. 6.8%; *p* < 0.01).

χ^2^ analysis showed a higher infection rate in patients with construct anchorage in metastatically involved adjacent vertebrae compared with those without (26.7% vs. 6.3%, p = 0.0035; Fig. 2). Additional associations were observed for intraoperative cerebrospinal fluid leakage (75%, p = 0.0004), thoracic spine involvement (14.3% vs. 4.2%, p = 0.0370), and hypertension (15.6% vs. 5.3%, p = 0.0411).

Univariate regression analysis (Table 2) confirmed increased odds of wound infection associated with adjacent level instrumentation (OR 4.78, p = 0.0044), ACE-27 comorbidity score of 1 (OR 3.54, p = 0.0360), and implant failure (OR 4.88, p = 0.038).

Exploratory multivariable logistic regression was performed to assess adjusted associations; results are shown in Supplemental Table S3 and Fig. 4B and are interpreted cautiously given the limited number of failure events.

## 7 Associations with postoperative bleeding

Postoperative bleeding or hematoma occurred in 12 patients (8.1%).

**Fig. 3:**
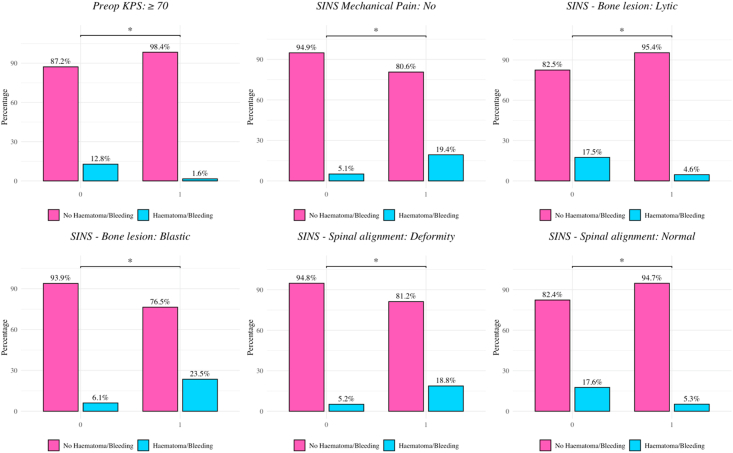
**Patient and disease characteristics significantly associated with postoperative bleeding/hematoma (p < 0.05).** Bar plots illustrating significant associations between baseline variables and the occurrence of postoperative hematoma or bleeding (p < 0.05, Chi-square test). Pink bars indicate patients without hematoma/bleeding, blue bars those with the complication, with percentages shown for each group. In the top left panel, a preoperative Karnofsky Performance Score (KPS) ≥ 70 was associated with a significantly lower bleeding risk (1.6% vs. 12.8%; p < 0.05). The top middle panel shows that absence of mechanical pain (SINS) was linked to a lower complication rate (5.1%) compared to those with pain (19.4%) (p < 0.05). The top right panel demonstrates that lytic bone lesions were associated with fewer hematoma/bleeding events (4.6% vs. 17.5%; p < 0.05). In the bottom left panel, patients with osteoblastic bone lesions had a higher bleeding rate (23.5%) than those without (6.1%) (p < 0.05). The bottom middle panel indicates that spinal deformity was significantly associated with increased bleeding risk (18.8% vs. 5.2%; p < 0.05). The bottom right panel shows that patients with normal spinal alignment (SINS) experienced fewer bleeding complications (5.3%) compared to those without normal alignment (17.6%) (p < 0.05).

On χ^2^ testing, higher bleeding rates were observed in patients with osteoblastic lesions, spinal deformity, and mechanical pain, whereas lower rates were observed in those with lytic lesions, normal spinal alignment, and preoperative Karnofsky Performance Scores ≥70 (all *p* < 0.05; Fig. 3).

**Fig. 4:**
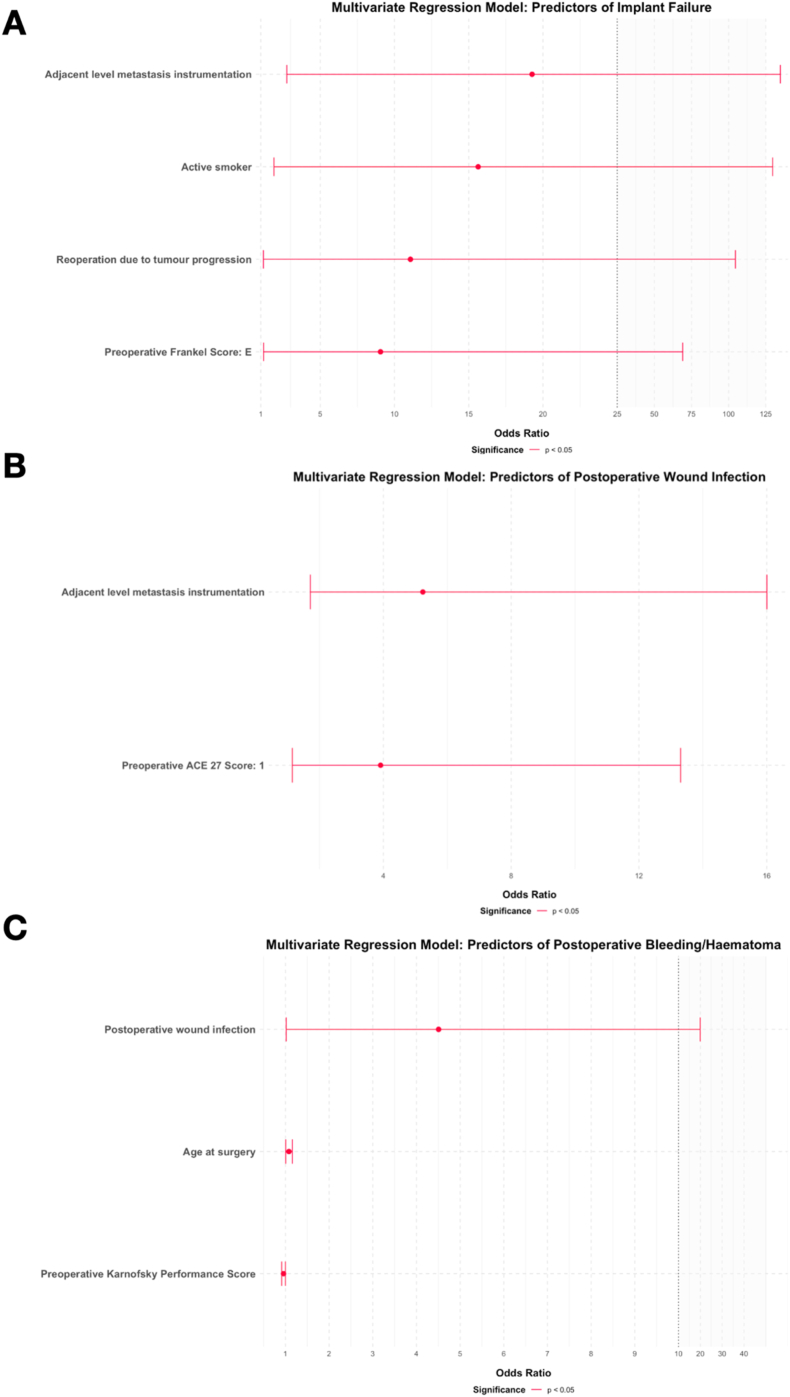
**Odds ratio plots from the exploratory multivariable regression analysis examining associations with implant failure (A), wound infection (B) and bleeding/hematoma (C).** The plot displays odds ratios with 95% confidence intervals derived from the multivariable logistic regression model assessing factors associated with implant failure (**A**), postoperative wound infection (**B**) or postoperative bleeding or hematoma (**C**) following stabilization surgery for spinal metastases. Only variables retained in the final exploratory model and reaching statistical significance (p < 0.05) are shown and highlighted in red. **A**, Anchoring the cranial or caudal end of the construct in metastatically involved adjacent vertebrae was associated with the highest odds of implant failure (OR = 19.27, 95% CI: 2.75–64.04), followed by repeat surgery at the same spinal level due to tumor progression (OR = 11.07, 95% CI: 1.17–104.57), preoperative Frankel Score E (OR = 9.06, 95% CI: 1.19–69.04), and active smoking (OR = 15.63, 95% CI: 1.88–129.69). For visualization purposes, the x-axis is truncated beyond an odds ratio of 25 to enhance interpretability while preserving the relative magnitude of effects. **B**, Instrumentation anchored in adjacent metastatically involved vertebrae was associated with higher odds of postoperative wound infection (OR = 5.23, 95% CI: 1.71–16.00). In addition, a preoperative ACE-27 comorbidity score of 1 was independently associated with an increased risk of wound infection (OR = 3.92, 95% CI: 1.15–13.30). **C**, Postoperative wound infection was associated with higher odds of bleeding or hematoma (OR = 4.51, 95% CI: 1.02–19.94). Increasing age at surgery was also associated with elevated bleeding risk (OR = 1.08, 95% CI: 1.01–1.16). In contrast, a higher preoperative Karnofsky Performance Score was associated with lower odds of postoperative bleeding or hematoma (OR = 0.96, 95% CI: 0.92–0.99).

Univariate regression (Table 3) identified wound infection (OR 5.21, *p* = 0.0157), advanced age at surgery (OR 1.09, *p* = 0.0104), impaired preoperative mobility (OR 3.78, *p* = 0.0308), and lower postoperative Karnofsky Performance Score (OR 0.96, *p* = 0.0100) as significantly associated with postoperative bleeding.

**Table 3:** Univariate regression model for post-operative bleeding/hematoma (p < 0.05).

*Variable*	Coefficient	Odds Ratio	CI: Lower	CI: Upper	*p-*Value
*Postoperative Karnofsky Performance Score in percentage*	−0.04	0.96	0.93	0.99	0.0100
*Age at surgery*	0.09	1.09	1.03	1.18	0.0104
*Age at diagnosis of spinal metastases*	0.08	1.09	1.02	1.17	0.0144
*Postoperative wound infection*	1.65	5.21	1.25	19.33	0.0157
*Neurological improvement after surgery*	1.89	6.61	1.64	44.07	0.0173
*Preoperative Karnofsky Performance Score*	−0.05	0.96	0.92	0.99	0.0251
*Mobility level before surgery: assistance*	1.33	3.78	1.14	13.49	0.0308
*Preoperative Karnofsky ≥ 70*	−2.21	0.11	0.06	0.59	0.0367

Table 3 presents the results of the univariate logistic regression analyses examining associations with postoperative bleeding or hematoma following stabilization surgery for spinal metastases. Neurological improvement after surgery was associated with higher odds of postoperative bleeding or hematoma (OR = 6.61, 95% CI: 1.64–44.07, p = 0.0173). Postoperative wound infection was also associated with an increased risk of bleeding or hematoma (OR = 5.21, 95% CI: 1.25–19.33, p = 0.0157). Preoperative mobility requiring assistance was associated with higher bleeding risk (OR = 3.78, 95% CI: 1.14–13.49, p = 0.0308), as were higher age at surgery (OR = 1.09, 95% CI: 1.03–1.18, p = 0.0104) and age at diagnosis of spinal metastases (OR = 1.09, 95% CI: 1.02–1.17, p = 0.0144). In contrast, higher functional status was associated with lower odds of postoperative bleeding, including higher postoperative Karnofsky Performance Score (OR = 0.96, 95% CI: 0.93–0.99, p = 0.0100) and preoperative Karnofsky Performance Score ≥70 (OR = 0.11, 95% CI: 0.06–0.59, p = 0.0367). Variables evaluated but not significantly associated with postoperative bleeding or hematoma included demographic factors (sex, body mass index), smoking history (current or former), primary tumor type, metastatic burden (synchronous or metachronous bone metastases; visceral, pulmonary, hepatic, cerebral, adrenal, lymph node, soft tissue, and other distant metastases), number and anatomical distribution of vertebral body metastases, affected spinal regions (cervical, thoracic, lumbar, sacral), comorbidity burden (ACE-27 score and individual comorbidities), spinal instability before or after surgery, preoperative neurological status (Frankel grades, presence of ataxia), postoperative pain outcomes, surgical approach and technique, staged procedures, stabilization-only surgery, repeat spinal surgery, implant misplacement, and other surgery-related complications including dural leak or implant failure.

Exploratory multivariable logistic regression was performed to assess adjusted associations; results are shown in Supplemental Table S4 and Fig. 4C, and are interpreted cautiously given the limited number of failure events.

## 8 Non-significant associations

Supplemental Table S5 summarizes candidate variables evaluated in univariate analyses that were not statistically significant, including: demographic factors (age at diagnosis, age at surgery, sex, BMI, height, weight), tumor characteristics (primary tumor type, visceral and distant metastases, number of vertebral levels involved), comorbidities (ACE-27 score, organ-specific conditions), preoperative clinical status (Karnofsky score, neurological symptoms, Frankel grade, mobility level, ataxia), surgical approach and technique (type, method, staged procedures), and pain or neurological outcomes.

Notably, for implant failure, no significant associations were observed with spinal level, tumor type, comorbidity burden or surgical approach. Similarly, for wound infection, neither spinal level, tumor histology, visceral metastases, preoperative performance, and surgical strategy showed significant predictive value. For postoperative bleeding, spinal level, tumor type, comorbidity profile, surgical technique and preoperative functional status were not significant predictors.

## 9 Discussion

This study provides insight into factors associated with implant failure and postoperative complications following stabilization surgery for spinal metastases. Across analyses, anchoring the cranial or caudal end of the construct in vertebrae affected by metastases was consistently associated with an increased risk of implant failure, alongside reoperation for tumor progression and active smoking. Associations between postoperative bleeding, wound infection, advanced age, and lower preoperative functional status were also observed; however, these relationships likely reflect overlapping risk profiles and perioperative vulnerability rather than direct causal pathways. Importantly, construct anchorage in metastatically involved adjacent vertebrae was not itself associated with postoperative bleeding but was consistently associated with wound infection, suggesting that these complications may co-occur due to shared mechanical and biological risk factors.

Our findings underscore the importance of incorporating clinical and anatomical considerations alongside radiographic tools such as the Spinal Instability Neoplastic Score (SINS) when planning surgical stabilization. While SINS remains valuable for assessing baseline instability, it did not retain an association with implant failure after adjustment, suggesting that radiographic instability alone may be insufficient to capture the mechanical challenges of metastatic fixation. Particular attention should therefore be given to cases in which adjacent vertebrae are affected by metastases, as compromised bone quality at the construct ends may weaken the bone–screw interface and predispose to mechanical failure. This has direct implications for surgical planning, raising the question of whether constructs should be extended to anchor in uninvolved vertebrae when anatomically and oncologically feasible.

Biomechanically, instrumentation anchored in metastatic bone likely compromises construct stability due to reduced bone-screw interface strength - particularly in lytic lesions that erode trabecular architecture and weaken anchoring capacity (Kumar et al., 2021). In the absence of biological fusion, which is often deprioritized in metastatic spine surgery, mechanical loads are borne entirely by the hardware, increasing the risk of fatigue failure over time. Notably, most centers, including our own, do not routinely use graft material or attempt arthrodesis in this context. However, preclinical data suggest that interbody fusion cages may redistribute anterior load and reduce posterior implant stress (Xu et al., 2019). Moreover, combined anterior-posterior fixation has been shown to better balance load sharing across spinal columns, which may mitigate mechanical overload of posterior instrumentation. However, these load-sharing concepts should be interpreted cautiously in the setting of metastatic disease. Unlike degenerative spinal disorders, tumor-related instability is characterized by ongoing vertebral destruction and limited biological healing potential, such that instrumentation often functions primarily as a load-bearing rather than a fusion-supported construct. The applicability of biomechanical principles derived from degenerative surgery is therefore necessarily limited in this oncological context.

Tumor progression following the index procedure, often necessitating extended resections or construct elongation, likely imposes additional mechanical strain on an already vulnerable system. Active smoking was also associated with implant failure, consistent with its known adverse effects on bone vascularity, osteoblast function, and tissue regeneration (Schroeder et al., 2016a). Interestingly, patients with preoperative Frankel Score E, who are typically fully ambulatory, demonstrated higher rates of implant failure, potentially reflecting increased cyclical loading during early postoperative mobilization when mechanical stability depends entirely on instrumentation rather than fusion (Schroeder et al., 2016b).

Collectively, these findings suggest that implant failure in metastatic spinal stabilization is not merely a function of baseline radiographic instability, but rather a dynamic process governed by mechanical, biological, and patient-driven stressors that evolve postoperatively. Patient-related substrate factors likely contribute further to this risk profile. In particular, impaired bone quality, including osteoporosis or reduced bone mineral density, may further weaken the bone-implant interface in patients with metastatic disease, especially in the presence of osteolytic destruction. Likewise, frailty may influence both postoperative complication risk and the ability to tolerate mechanical stress after surgery. These variables were not systematically available in our retrospective dataset and therefore could not be analyzed directly, but they should be incorporated into future prospective studies.

Construct anchorage in metastatically involved adjacent vertebrae was also consistently associated with postoperative wound infection. Operating in tumor-infiltrated bone may increase surgical complexity, prolong operative time, and exacerbate soft tissue trauma, all of which may elevate infection risk. Compromised local tissue healing, particularly in the context of prior or subsequent radiation therapy and systemic oncological treatments, combined with the presence of instrumentation as a potential nidus for bacterial adherence, may further contribute to vulnerability. When anchoring in metastatic vertebrae is unavoidable, meticulous surgical technique and optimized perioperative management are therefore essential. Additional construct-related factors may also influence mechanical durability. Cement augmentation of pedicle screws may improve fixation in osteolytic bone, while construct length and junctional segment involvement may alter load distribution and susceptibility to mechanical complications. Similarly, minimally invasive and open techniques may differ with respect to soft-tissue preservation, construct characteristics, and the biological environment for healing. Because these variables were not systematically available in the present dataset, their influence could not be evaluated directly.

Notably, a broad range of clinically plausible variables - including spinal level, tumor histology, comorbidity burden, and surgical approach - were evaluated but did not demonstrate significant associations with implant failure, wound infection, or postoperative bleeding. While these findings may in part reflect cohort-specific characteristics or limited statistical power, they may also suggest that the mechanical and biological consequences of anchoring constructs in metastatic bone outweigh many traditionally assumed risk factors.

Overall, these results provide clinically relevant guidance for surgical planning in spinal metastasis, highlighting the importance of construct anchorage strategy and supporting efforts to avoid fixation in metastatic vertebrae whenever feasible, extend constructs into structurally uninvolved bone, and consider augmentation strategies in cases of diffuse skeletal disease to potentially reduce postoperative complications.

## 10 Limitations

This study has several limitations. First, as a single-center retrospective analysis, the generalizability of the findings to other institutions and practice settings may be limited. Second, the relatively small number of outcome events, particularly implant failure and wound infection, restricts statistical power for subgroup analyses and limits the robustness of multivariable modeling. Accordingly, multivariable analyses were interpreted as exploratory and complementary rather than definitive.

Metastatic involvement at construct anchoring levels was classified as a binary variable based on imaging evidence of infiltration, without grading lesion morphology or extent of osseous destruction, which may introduce heterogeneity in biomechanical risk. Correlation with the degree of vertebral destruction or tumour volume was not possible and may be important in future biomechanical risk stratification. In addition, objective measures of bone quality, such as bone mineral density, and standardized frailty indices were not consistently available and could not be evaluated. Furthermore, implant- and technique-related factors known to influence mechanical outcomes, including construct stiffness, cement augmentation, implant design, exact construct length, junctional segment involvement, and minimally invasive versus open instrumentation techniques, were not systematically recorded and could not be analyzed.

Residual confounding remains possible, especially for factors not captured in sufficient detail, such as specific systemic oncological therapies, timing and dose of perioperative radiation therapy, corticosteroid exposure, and nuanced surgical technique variables. In addition, implant-related factors known to influence mechanical outcomes, including construct stiffness, cement augmentation, and implant design, were not systematically recorded and could not be analyzed. Finally, the lack of long-term follow-up limits insight into late mechanical failures and the durability of stabilization strategies.

Future multicenter, prospective studies with standardized surgical, oncological, and follow-up protocols are needed to validate these findings, better characterize temporal risk patterns, and refine complication-aware surgical decision-making in patients with spinal metastases.

## 11 Conclusion

This study shows that implant failure and postoperative complications following spinal stabilization for metastatic disease are associated with a combination of mechanical, biological, and patient-related factors. In particular, anchoring the cranial or caudal end of fixation constructs in vertebrae affected by metastases was consistently associated with higher rates of implant failure and postoperative wound infection, alongside tumor progression and active smoking.

These findings suggest that, when surgically feasible, avoidance of construct anchorage in metastatic vertebrae, extension of fixation into structurally uninvolved bone, and consideration of adjunctive strategies such as cement augmentation in cases of diffuse disease may help reduce the risk of postoperative complications. Further prospective, multicenter studies are warranted to validate these associations and refine complication-aware surgical strategies in patients with spinal metastases.

## Ethical approval

Approved by the Ethics Committee of the Landesärztekammer Rheinland-Pfalz (no. 2021-16187). Informed consent was obtained from all subjects or their legal guardian.

## Declaration of competing interest

The authors declare that they have no known competing financial interests or personal relationships that could have appeared to influence the work reported in this paper.
