# Supplementary material for: Implant failure and postoperative complications after stabilization surgery for spinal metastases: A single-center cohort study
Source: Brain Spine. 2026 May 9;6:106088. doi: 10.1016/j.bas.2026.106088 (PMC13196448; doi:10.1016/j.bas.2026.106088)
Supplement: Multimedia component 1 [file mmc1.docx]

**Supplementary Tables**

**Implant Failure and Postoperative Complications After Stabilization Surgery for Spinal Metastases:**

**A Single-Center Cohort Study**

Santhosh G. Thavarajasingam, MBBS ^1,2^ ; Ahmed Salih, BSc^2^ ; Christine Brühl^1^ ;

Daniel Scurtu^3^; Sree Kanakala^2,4^ ; Srikar Namireddy^2,4^ ; Daniele Ramsay, MBBS^2^ ;

Ahkash Thavarajasingam^2^ ; Tim-Mathis Beutel^2^ ; Jonathan Neuhoff, MD^5,6^ ;

Josephine Pollok^3^ ; Merih Turgut^3^ ; Dragan Jankovic, MD^1^ ;

Florian Ringel, MD^1^ ; Andreas Kramer, MD*^1^

**INSTITUTIONS:**

1. Department of Neurosurgery, LMU University Hospital, LMU Munich, Germany.
2. Imperial Brain & Spine Initiative, Imperial College London, United Kingdom.
3. Department of Neurosurgery, University Medical Center Mainz, Germany.
4. Faculty of Medicine, Imperial College London, United Kingdom
5. Center for Spinal Surgery and Neurotraumatology, Berufsgenossenschaftliche Unfallklinik Frankfurt am Main, Frankfurt, Germany.
6. Spondylodiscitis Study Group, EANS Spine Section, Hamburg, Germany.

| Variable | | Summary Statistic | |
| --- | --- | --- | --- |
|  |  |  | |
| Age (yrs. mean ± SD) | | 65.1 ± 11.6 | |
| Male (n, %) | | 97 (65.1 %) | |
| Co-morbidities (n, %) | |  | |
|  | Diabetes | 21 (14.1%) | |
|  | COPD | 6 (4.0%) | |
|  | Renal Disease | 8 (5.4%) | |
|  | Cardiovascular Disease | 27 (18.1%) | |
| Number of Bony Metastasis (n, %) | |  | |
|  | 1 metastasis | 41 (27.5%) | |
|  | 2-3 metastasis | 42 (28.2%) | |
|  | >3 metastasis | 65 (43.6%) | |
| Surgery Indications (n, %) | |  | |
|  | Pain | 17 (11.4%) | |
|  | Instability | 23 (15.4%) | |
|  | Neurology Deficit | 67 (45.0%) | |
| Karnofsky score Before Surgery (Median ± Median Absolute Deviation) | | 60 ± 10 | |
| Frankel grading Before Surgery | |  | |
|  | A | 10 (6.7%) | |
|  | B | 2 (1.3%) | |
|  | C | 21 (14.1%) | |
|  | D | 52 (34.9%) | |
|  | E | 63 (42.3 %) | |
| Primary Tumor (n, %) | |  | |
|  | Liver | 8 (5.4%) | |
|  | Lung | 36 (24.2%) | |
|  | Prostate | 26 (17.4%) | |
|  | Thyroid | 6 (4.0%) | |
|  | Kidney | 13 (8.7%) | |
|  | Breast | 21 (14.1%) | |
| Surgical Modalities (n, %) | |  | |
|  | Corpectomy | 60(40.3 %) | |
|  | Laminectomy | 97 (65.1 %) | |
|  | Kyphoplasty | 3(2.0 %) | |
| Improvement in neurological symptoms | | 20 (13.4%) | |
| Karnofsky score Post-surgery  (Median ± Median Absolute Deviation) | | 70 ± 10 | |
| Frankel grading Post-surgery | |  | |
|  | A | 7 (4.7%) | |
|  | B | 3 (2.0%) | |
|  | C | 9 (14.1%) | |
|  | D | 52 (34.9%) | |
|  | E | 77 (51.7%) | |
| Outcomes (n, %) | |  | |
|  | Implant Failure | 9 (6.0%) | |
|  | Wound healing disorders  Postoperative hematoma | 16(10.7 %)  12 (8.1 %) | |
|  | Mortality immediately following surgery | | 2 (1.3%) |
|  | Overall Mortality | | 115 (77.2 %) |

**Supplemental Table S1: Summary of patient and disease characteristics of the study population.**

Supplemental Table S1 shows a summary of Study Population: This table summarizes the demographic, clinical, and surgical characteristics of the study population (n = 149). COPD, Chronic Obstructive Pulmonary Disease; SD, Standard Deviation; n, Number of patients.

| Variable | Coefficient | Std. Error | Odds Ratio | CI: Lower | CI: Upper | *p*-Value |
| --- | --- | --- | --- | --- | --- | --- |
| *Adjacent level metastasis instrumentation* | 2.96 | 0.99 | 19.27 | 2.75 | 64.04 | 0.0029 |
| *Reoperation at the same spinal level due to tumor progression* | 2.40 | 1.15 | 11.1 | 1.17 | 104.57 | 0.036 |
| *Preoperative Frankel Score: E* | 2.20 | 1.04 | 9.06 | 1.19 | 69.04 | 0.033 |
| *Active smoker* | 2.75 | 1.08 | 15.63 | 1.88 | 129.69 | 0.011 |

**Supplemental Table S2: Multivariable regression model for implant failure.**

Supplemental Table S2 presents the results of the exploratory multivariable logistic regression analysis examining adjusted associations with implant failure following stabilization surgery for spinal metastases. After adjustment for selected clinically relevant covariates, anchoring the cranial or caudal end of the construct in metastatically involved vertebrae remained strongly associated with implant failure (OR = 19.27, 95% CI: 2.75–64.04, p = 0.0029). Reoperation at the same spinal level due to tumor progression was also associated with higher odds of implant failure (OR = 11.07, 95% CI: 1.17–104.57, p = 0.036). In addition, a preoperative Frankel Score of E (OR = 9.06, 95% CI: 1.19–69.04, p = 0.033) and active smoking (OR = 15.63, 95% CI: 1.88–129.69, p = 0.011) remained associated with implant failure in the multivariable model.

| Variable | Coefficient | Std. Error | Odds Ratio | CI: Lower | CI: Upper | *p*-Value |
| --- | --- | --- | --- | --- | --- | --- |
| *Adjacent level metastasis instrumentation* | 0.57 | 0.57 | 5.23 | 1.71 | 16.00 | 0.0037 |
| *Preoperative ACE 27 Score: 1* | 1.36 | 0.62 | 3.92 | 1.15 | 13.30 | 0.0290 |

**Supplemental Table S3: Multivariable regression model for postoperative wound infections.**

Supplemental Table S3 presents the results of the exploratory multivariable logistic regression analysis examining adjusted associations with postoperative wound infection following stabilization surgery for spinal metastases. After adjustment for selected clinically relevant covariates, adjacent level metastasis instrumentation remained associated with postoperative wound infection (OR = 5.23, 95% CI: 1.71–16.00, p = 0.0037). A preoperative ACE-27 comorbidity score of 1 was also associated with an increased risk of wound infection in the multivariable model (OR = 3.92, 95% CI: 1.15–13.30, p = 0.0290).

| Variable | Coefficient | Std. Error | Odds Ratio | CI: Lower | CI: Upper | *p*-Value |
| --- | --- | --- | --- | --- | --- | --- |
| *Age at surgery* | 0.08 | 0.04 | 1.08 | 1.01 | 1.16 | 0.0315 |
| *Postoperative wound infection* | 1.51 | 0.76 | 4.51 | 1.02 | 19.94 | 0.0472 |
| *Postoperative KPS* | -0.04 | 0.02 | 0.96 | 0.92 | 1.00 | 0.0468 |

**Supplemental Table S4: Multivariable regression model for postoperative post-operative bleeding/hematoma (p<0.05).**

Supplemental Table S4 presents the results of the exploratory multivariable logistic regression analysis examining adjusted associations with postoperative bleeding or hematoma following stabilization surgery for spinal metastases. After adjustment for selected clinically relevant covariates, postoperative wound infection remained associated with an increased risk of postoperative bleeding or hematoma (OR = 4.51, 95% CI: 1.02–19.94, p = 0.0472). Increasing age at surgery was also associated with higher odds of postoperative bleeding (OR = 1.08, 95% CI: 1.01–1.16, p = 0.0315). In contrast, a higher preoperative Karnofsky Performance Score was associated with lower odds of postoperative bleeding or hematoma (OR = 0.96, 95% CI: 0.92–1.00, p = 0.0468).

| Variable | Coefficient | Odds Ratio | CI: Lower | CI: Upper | *p*-Value |
| --- | --- | --- | --- | --- | --- |
| Outcome: Implant failure ~ |  |  |  |  |  |
| *Age at diagnosis of spinal metastases* | -0.0283 | 0.9721 | 0.9218 | 1.028600e+00 | 0.3042 |
| *Age at surgery* | -0.0237 | 0.9765 | 0.9255 | 1.034600e+00 | 0.3967 |
| *Time between diagnosis of the spinal metastases and surgery* | 0.0009 | 1.0009 | 0.9996 | 1.002000e+00 | 0.0994 |
| *Female sex* | 0.4274 | 1.5333 | 0.3650 | 6.053600e+00 | 0.5380 |
| *Type of tumor: Prostate* | -17.0271 | 0.0000 | NA | 6.171500e+69 | 0.9936 |
| *Type of tumor: Lung* | -0.9808 | 0.3750 | 0.0199 | 2.148800e+00 | 0.3631 |
| *Type of tumor: Breast* | -0.2877 | 0.7500 | 0.0393 | 4.416500e+00 | 0.7914 |
| *Type of tumor: Kidney* | 1.2092 | 3.3506 | 0.4623 | 1.602840e+01 | 0.1603 |
| *Type of tumor: Liver* | -14.8805 | 0.0000 | NA | 1.350288e+44 | 0.9915 |
| *Type of tumor: Colorectal* | 1.0266 | 2.7917 | 0.1391 | 1.935220e+01 | 0.3677 |
| *Type of tumor: Esophagus* | -14.8655 | 0.0000 | NA | 1.963485e+60 | 0.9927 |
| *Type of tumor: Urothelial* | -13.8433 | 0.0000 | NA | 1.891533e+72 | 0.9920 |
| *Type of tumor: Thyroid* | 1.2164 | 3.3750 | 0.1659 | 2.456150e+01 | 0.2920 |
| *Type of tumor: Other* | 0.7949 | 2.2143 | 0.3120 | 1.015200e+01 | 0.3466 |
| *Synchronous bone metastases* | -1.9650 | 0.1402 | 0.0075 | 7.929000e-01 | 0.0673 |
| *Metachronous bone metastases* | 1.9650 | 7.1351 | 1.2613 | 1.341365e+02 | 0.0673 |
| *Visceral distant metastases* | 0.1946 | 1.2148 | 0.3091 | 5.085600e+00 | 0.7785 |
| *Lung metastases* | 0.5241 | 1.6889 | 0.4015 | 6.677700e+00 | 0.4507 |
| *Liver metastases* | -1.0186 | 0.3611 | 0.0191 | 2.067300e+00 | 0.3448 |
| *Brain metastases* | 0.1178 | 1.1250 | 0.0585 | 6.818400e+00 | 0.9145 |
| *Adrenal metastases* | -15.9350 | 0.0000 | NA | 6.116790e+54 | 0.9925 |
| *Distant lymph node metastases* | -1.1979 | 0.3018 | 0.0160 | 1.721200e+00 | 0.2659 |
| *Soft tissue metastases* | 0.8377 | 2.3111 | 0.5465 | 9.198300e+00 | 0.2301 |
| *Other distant metastases* | -0.5381 | 0.5839 | 0.0847 | 2.529800e+00 | 0.5125 |
| *Number of vertebral body metastases at time of surgery: 2 to 3* | 0.0870 | 1.0909 | 0.2223 | 4.336900e+00 | 0.9051 |
| *Number of vertebral body metastases at time of surgery: more than 3* | -0.8770 | 0.4160 | 0.0605 | 1.795200e+00 | 0.2849 |
| *Cervical bone lesion in surgery* | 0.0215 | 1.0217 | 0.2083 | 4.057300e+00 | 0.9765 |
| *Thoracic bone lesion in surgery* | 0.0764 | 1.0794 | 0.2465 | 7.480900e+00 | 0.9263 |
| *Lumbar bone lesion in surgery* | -0.5500 | 0.5769 | 0.1181 | 2.278300e+00 | 0.4493 |
| *Sacral bone lesion in surgery* | 0.0408 | 1.0417 | 0.0542 | 6.272600e+00 | 0.9703 |
| *Karnofsky Performance Score before first surgery* | 0.0133 | 1.0134 | 0.9698 | 1.064100e+00 | 0.5661 |
| *Karnofsky Performance Score before first surgery: 70 or over 70 KPS* | 0.5694 | 1.7672 | 0.4493 | 7.407400e+00 | 0.4108 |
| *Preoperative Comorbidity ACE 27 Score* | 0.0167 | 1.0168 | 0.4025 | 2.287600e+00 | 0.9695 |
| *Preoperative Comorbidity ACE 27 Score: 1* | 0.2803 | 1.3235 | 0.3368 | 5.541100e+00 | 0.6853 |
| *Preoperative Comorbidity ACE 27 Score: 2* | -15.9758 | 0.0000 | NA | 2.978171e+46 | 0.9913 |
| *Preoperative Comorbidity ACE 27 Score: 3* | 1.2164 | 3.3750 | 0.1659 | 2.456150e+01 | 0.2920 |
| *Comorbidity: Renal disease* | -14.8805 | 0.0000 | NA | 1.350289e+44 | 0.9915 |
| *Comorbidity: Cardiovascular disease* | -0.6013 | 0.5481 | 0.0289 | 3.179200e+00 | 0.5786 |
| *Comorbidity: High blood pressure* | -0.3089 | 0.7342 | 0.1754 | 2.885600e+00 | 0.6552 |
| *Comorbidity: Peripheral arterial disease* | 1.0266 | 2.7917 | 0.1391 | 1.935220e+01 | 0.3677 |
| *Comorbidity: Depression* | -14.8655 | 0.0000 | NA | 1.963485e+60 | 0.9927 |
| *Comorbidity: Polyneuropathy/Chronic neuromuscular disorder* | -14.8655 | 0.0000 | NA | 1.963485e+60 | 0.9927 |
| *Comorbidity: Rheumatism* | -12.8288 | 0.0000 | NA | 1.543062e+123 | 0.9930 |
| *Comorbidity: Prothrombotic/haematological* | -12.8288 | 0.0000 | NA | 1.543062e+123 | 0.9930 |
| *Comorbidity: Diabetes* | -0.2877 | 0.7500 | 0.0393 | 4.416500e+00 | 0.7914 |
| *Comorbidity: COPD* | 1.2164 | 3.3750 | 0.1659 | 2.456150e+01 | 0.2920 |
| *Comorbidity: Liver* | -14.8580 | 0.0000 | NA | 8.196051e+72 | 0.9933 |
| *Comorbidity: Epilepsy* | -12.8288 | 0.0000 | NA | 1.543062e+123 | 0.9930 |
| *Comorbidity: Obesity* | 0.6491 | 1.9138 | 0.3859 | 7.725800e+00 | 0.3786 |
| *Comorbidity: Stroke* | -14.8580 | 0.0000 | NA | 8.196051e+72 | 0.9933 |
| *Comorbidity: Asthma* | -13.8433 | 0.0000 | NA | 1.891533e+72 | 0.9920 |
| *Comorbidity: Bronchitis* | -13.8433 | 0.0000 | NA | 1.891533e+72 | 0.9920 |
| *Comorbidity: Ulcerative colitis* | -12.8288 | 0.0000 | NA | 1.543062e+123 | 0.9930 |
| *Comorbidity: Pulmonary embolism* | -13.8360 | 0.0000 | NA | 3.080770e+109 | 0.9935 |
| *Comorbidity: Sleep apnoea* | -12.8288 | 0.0000 | NA | 1.543062e+123 | 0.9930 |
| *Comorbidity: Hypothyroidism* | -13.8360 | 0.0000 | NA | 3.080770e+109 | 0.9935 |
| *Comorbidity: Dementia* | -13.8433 | 0.0000 | NA | 1.891533e+72 | 0.9920 |
| *Comorbidity: Parkinson’s disease* | -12.8288 | 0.0000 | NA | 1.543062e+123 | 0.9930 |
| *Comorbidity: Psoriasis* | -12.8288 | 0.0000 | NA | 1.543062e+123 | 0.9930 |
| *Smoker previously* | 0.0895 | 1.0936 | 0.1573 | 4.816500e+00 | 0.9140 |
| *Height in cm* | -0.0573 | 0.9443 | 0.8596 | 1.026500e+00 | 0.2013 |
| *Weight in kg* | -0.0117 | 0.9883 | 0.9461 | 1.027100e+00 | 0.5737 |
| *BMI* | -0.0038 | 0.9962 | 0.8646 | 1.136300e+00 | 0.9560 |
| *Instability before surgery: yes* | 0.0895 | 1.0936 | 0.1573 | 4.816500e+00 | 0.9140 |
| *Instability before surgery: potential* | 0.4393 | 1.5515 | 0.3575 | 1.070140e+01 | 0.5933 |
| *Instability before surgery: no* | -15.9191 | 0.0000 | NA | 2.419610e+59 | 0.9930 |
| *Frankel Neuro Classification before surgery* | -0.4668 | 0.6270 | 0.2273 | 1.249400e+00 | 0.2722 |
| *Frankel Neuro Classification before surgery: D* | -1.5226 | 0.2181 | 0.0116 | 1.237800e+00 | 0.1567 |
| *Frankel Neuro Classification before surgery: C* | -15.9842 | 0.0000 | NA | 1.505084e+45 | 0.9910 |
| *Frankel Neuro Classification before surgery: B* | -13.8360 | 0.0000 | NA | 3.080770e+109 | 0.9935 |
| *Frankel Neuro Classification before surgery: A* | 0.5985 | 1.8194 | 0.0929 | 1.165050e+01 | 0.5915 |
| *Mobility level before surgery: independent* | 0.1660 | 1.1806 | 0.3004 | 4.942300e+00 | 0.8104 |
| *Mobility level before surgery: assistance* | 0.1884 | 1.2073 | 0.2457 | 4.809200e+00 | 0.7966 |
| *Mobility level before surgery: immobile* | -0.6013 | 0.5481 | 0.0289 | 3.179200e+00 | 0.5786 |
| *Ataxia before surgery: yes* | -0.2509 | 0.7781 | 0.2504 | 1.874500e+00 | 0.6106 |
| *Ataxia after surgery; yes* | -0.3346 | 0.7156 | 0.2014 | 1.739000e+00 | 0.5186 |
| *Neurological improvement after surgery* | -0.5787 | 0.5606 | 0.1147 | 2.213600e+00 | 0.4260 |
| *Surgical type: dorsal* | -0.2584 | 0.7723 | 0.1936 | 3.799500e+00 | 0.7240 |
| *Surgical type: ventral* | 0.1178 | 1.1250 | 0.0585 | 6.818400e+00 | 0.9145 |
| *Surgical type: ventrodorsal/360°* | 0.2733 | 1.3143 | 0.1883 | 5.833000e+00 | 0.7424 |
| *Surgical method: spondylodesis* | -0.0408 | 0.9600 | 0.1594 | 1.843690e+01 | 0.9703 |
| *Surgical method: laminectomy* | -0.4274 | 0.6522 | 0.1652 | 2.739900e+00 | 0.5380 |
| *Surgical method: kypho- or vertebroblasty* | -13.8433 | 0.0000 | NA | 1.891533e+72 | 0.9920 |
| *Surgical method: corpectomy* | 1.1585 | 3.1852 | 0.8052 | 1.559010e+01 | 0.1116 |
| *Staged surgery: yes* | 0.5232 | 1.6875 | 0.3413 | 6.782200e+00 | 0.4766 |
| *Only stabilisation* | 0.3646 | 1.4400 | 0.3430 | 5.680300e+00 | 0.5991 |
| *Postoperative Karnofsky Performance Score in percentage* | -0.0085 | 0.9915 | 0.9565 | 1.031800e+00 | 0.6541 |
| *Pain postoperative: better* | -0.0541 | 0.9474 | 0.2384 | 4.647700e+00 | 0.9410 |
| *Pain postoperative: worse* | -13.8433 | 0.0000 | NA | 1.891533e+72 | 0.9920 |
| *Pain postoperative: same* | 0.2231 | 1.2500 | 0.2542 | 4.983000e+00 | 0.7603 |
| *Response to OP pain: partial* | -0.9359 | 0.3923 | 0.0571 | 1.691800e+00 | 0.2537 |
| *Response to OP pain: complete* | 0.8377 | 2.3111 | 0.5465 | 9.198300e+00 | 0.2301 |
| *Response to OP pain: worse* | -13.8433 | 0.0000 | NA | 1.891533e+72 | 0.9920 |
| *Other surgery related complications: implant misplacement* | -14.8655 | 0.0000 | NA | 1.963485e+60 | 0.9927 |
| Outcome: Wound infection ~ |  |  |  |  |  |
| *Age at diagnosis of spinal metastases* | 0.0245 | 1.024800e+00 | 0.9789 | 1.077800e+00 | 0.3163 |
| *Age at surgery* | 0.0294 | 1.029800e+00 | 0.9827 | 1.084900e+00 | 0.2418 |
| *Time between diagnosis of the spinal metastases and the surgery* | 0.0007 | 1.000700e+00 | 0.9996 | 1.001700e+00 | 0.1581 |
| *Female sex* | -0.1843 | 8.317000e-01 | 0.2497 | 2.434700e+00 | 0.7461 |
| *Type of tumor: Prostate* | -0.4326 | 6.488000e-01 | 0.0978 | 2.530600e+00 | 0.5834 |
| *Type of tumor: Lung* | 0.4025 | 1.495600e+00 | 0.4435 | 4.457800e+00 | 0.4854 |
| *Type of tumor: Breast* | -0.1542 | 8.571000e-01 | 0.1282 | 3.404500e+00 | 0.8463 |
| *Type of tumor: Kidney* | -0.3972 | 6.722000e-01 | 0.0357 | 3.798800e+00 | 0.7121 |
| *Type of tumor: Liver* | 0.1823 | 1.200000e+00 | 0.0620 | 7.428600e+00 | 0.8688 |
| *Type of tumor: Colorectal* | 0.3444 | 1.411100e+00 | 0.0722 | 9.066200e+00 | 0.7572 |
| *Type of tumor: Esophagus* | 0.5345 | 1.706700e+00 | 0.0861 | 1.155620e+01 | 0.6359 |
| *Type of tumor: Urothelial* | 1.4740 | 4.366700e+00 | 0.1961 | 4.829330e+01 | 0.2401 |
| *Type of tumor: Thyroid* | 1.5276 | 4.607100e+00 | 0.6009 | 2.593010e+01 | 0.0934 |
| *Type of tumor: Other* | -16.5937 | 0.000000e+00 | NA | 6.029640e+36 | 0.9914 |
| *Synchronous Bone metastases* | -0.3450 | 7.082000e-01 | 0.2295 | 2.019700e+00 | 0.5266 |
| *Metachronous bone metastases* | 0.3450 | 1.412000e+00 | 0.4951 | 4.357800e+00 | 0.5266 |
| *Visceral distant Metastases* | 0.5259 | 1.691900e+00 | 0.5936 | 5.220600e+00 | 0.3344 |
| *Lung metastases* | 0.2278 | 1.255800e+00 | 0.4045 | 3.611100e+00 | 0.6780 |
| *Liver metastases* | 0.3609 | 1.434700e+00 | 0.4260 | 4.268500e+00 | 0.5311 |
| *Brain metastases* | -16.5680 | 0.000000e+00 | NA | 3.136624e+39 | 0.9922 |
| *Adrenal metastases* | -0.5680 | 5.667000e-01 | 0.0302 | 3.145500e+00 | 0.5958 |
| *Distant Lymph node Metastases* | 0.7783 | 2.177800e+00 | 0.7292 | 6.289100e+00 | 0.1503 |
| *Soft tissue metastases* | 0.5579 | 1.747100e+00 | 0.5585 | 5.074900e+00 | 0.3133 |
| *Other distant Metastases* | 0.5569 | 1.745300e+00 | 0.5876 | 5.005700e+00 | 0.3004 |
| *Number of vertebral body metastases at time of surgery: 2 to 3* | 0.5924 | 1.808300e+00 | 0.6083 | 5.191600e+00 | 0.2711 |
| *Number of vertebral body metastases at time of surgery at time of surgery: more than 3* | 0.7581 | 2.134300e+00 | 0.7497 | 6.313900e+00 | 0.1563 |
| *Cervical bone lesion in surgery* | -0.4279 | 6.519000e-01 | 0.1745 | 1.993000e+00 | 0.4799 |
| *Thoracic bone lesion in surgery* | 1.6393 | 5.151500e+00 | 0.9860 | 9.488160e+01 | 0.1191 |
| *Lumbar bone lesion in surgery* | 0.1961 | 1.216700e+00 | 0.4239 | 3.493600e+00 | 0.7111 |
| *Sacral bone lesion in surgery* | 1.2123 | 3.361100e+00 | 0.8409 | 1.149910e+01 | 0.0629 |
| *Karnofsky Performance Score before first surgery* | -0.0036 | 9.964000e-01 | 0.9637 | 1.031400e+00 | 0.8352 |
| *Karnofsky Performance Score before first surgery: 70 or over 70 KPS* | -0.2231 | 8.000000e-01 | 0.2591 | 2.283600e+00 | 0.6824 |
| *Comorbidity ACE 27 Score before surgery* | -0.0076 | 9.924000e-01 | 0.4924 | 1.877000e+00 | 0.9820 |
| *Comorbidity ACE 27 Score before surgery: 2* | -0.9163 | 4.000000e-01 | 0.0215 | 2.159400e+00 | 0.3883 |
| *Comorbidity ACE 27 Score before surgery: 3* | -15.4945 | 0.000000e+00 | NA | 2.153332e+60 | 0.9923 |
| *Comorbidity: Renal disease* | -15.5103 | 0.000000e+00 | NA | 3.101853e+44 | 0.9912 |
| *Comorbidity: Cardiovascular disease* | -1.2933 | 2.744000e-01 | 0.0148 | 1.451300e+00 | 0.2204 |
| *Comorbidity: High blood pressure* | 1.1437 | 3.138500e+00 | 1.0339 | 1.167810e+01 | 0.0578 |
| *Comorbidity: Peripheral arterial disease* | 0.3444 | 1.411100e+00 | 0.0722 | 9.066200e+00 | 0.7572 |
| *Comorbidity: Depression* | -15.4945 | 0.000000e+00 | NA | 2.153332e+60 | 0.9923 |
| *Comorbidity: Polyneuropathy/Chronic neuromuscular disorder* | -15.4945 | 0.000000e+00 | NA | 2.153332e+60 | 0.9923 |
| *Comorbidity: Rheumatism* | -13.4559 | 0.000000e+00 | NA | 8.774805e+122 | 0.9926 |
| *Comorbidity: Prothrombotic/haematological* | -13.4559 | 0.000000e+00 | NA | 8.774805e+122 | 0.9926 |
| *Comorbidity: Diabetes* | -0.9764 | 3.767000e-01 | 0.0203 | 2.025700e+00 | 0.3574 |
| *Comorbidity: COPD* | 0.5345 | 1.706700e+00 | 0.0861 | 1.155620e+01 | 0.6359 |
| *Comorbidity: Liver* | -15.4866 | 0.000000e+00 | NA | 7.762856e+72 | 0.9930 |
| *Comorbidity: Epilepsy* | -13.4559 | 0.000000e+00 | NA | 8.774805e+122 | 0.9926 |
| *Comorbidity: Obesity* | -0.1892 | 8.276000e-01 | 0.1810 | 2.781400e+00 | 0.7789 |
| *Comorbidity: Stroke* | 0.7655 | 2.150000e+00 | 0.1062 | 1.576420e+01 | 0.5060 |
| *Comorbidity: Asthma* | -14.4711 | 0.000000e+00 | NA | 1.288780e+72 | 0.9917 |
| *Comorbidity: Bronchitis* | -14.4711 | 0.000000e+00 | NA | 1.288780e+72 | 0.9917 |
| *Comorbidity: Ulcerative colitis* | -13.4559 | 0.000000e+00 | NA | 8.774805e+122 | 0.9926 |
| *Comorbidity: Pulmonary embolism* | 2.1748 | 8.800000e+00 | 0.3365 | 2.304027e+02 | 0.1310 |
| *Comorbidity: Sleep apnoea* | -13.4559 | 0.000000e+00 | NA | 8.774805e+122 | 0.9926 |
| *Comorbidity: Hypothyroidism* | -14.4635 | 0.000000e+00 | NA | 2.031364e+109 | 0.9932 |
| *Comorbidity: Dementia* | -14.4711 | 0.000000e+00 | NA | 1.288780e+72 | 0.9917 |
| *Comorbidity: Parkinson’s disease* | -13.4559 | 0.000000e+00 | NA | 8.774805e+122 | 0.9926 |
| *Comorbidity: Psoriasis* | -13.4559 | 0.000000e+00 | NA | 8.774805e+122 | 0.9926 |
| *Smoker currently* | 0.3202 | 1.377400e+00 | 0.4094 | 4.091300e+00 | 0.5779 |
| *Smoker previously* | 0.2690 | 1.308600e+00 | 0.3448 | 4.101900e+00 | 0.6625 |
| *Height in cm* | -0.0206 | 9.796000e-01 | 0.9173 | 1.041700e+00 | 0.5230 |
| *Weight in kg* | -0.0085 | 9.916000e-01 | 0.9598 | 1.021300e+00 | 0.5899 |
| *BMI* | -0.0285 | 9.719000e-01 | 0.8702 | 1.078100e+00 | 0.5998 |
| *Instability before surgery: yes* | 0.2690 | 1.308600e+00 | 0.3448 | 4.101900e+00 | 0.6625 |
| *Instability before surgery: potential* | -0.3689 | 6.915000e-01 | 0.2396 | 2.152800e+00 | 0.5027 |
| *Instability before surgery: no* | -0.3972 | 6.722000e-01 | 0.0357 | 3.798800e+00 | 0.7121 |
| *Neurological symptoms before surgery* | -0.3185 | 7.273000e-01 | 0.2530 | 2.089000e+00 | 0.5479 |
| *Frankel Neuro Classification before surgery* | -0.3785 | 6.849000e-01 | 0.3460 | 1.148200e+00 | 0.2090 |
| *Frankel Neuro Classification before surgery: E* | 0.3494 | 1.418200e+00 | 0.4936 | 4.077500e+00 | 0.5098 |
| *Frankel Neuro Classification before surgery: D* | 0.1264 | 1.134800e+00 | 0.3661 | 3.255800e+00 | 0.8174 |
| *Frankel Neuro Classification before surgery: C* | -0.1542 | 8.571000e-01 | 0.1282 | 3.404500e+00 | 0.8463 |
| *Frankel Neuro Classification before surgery: B* | -14.4635 | 0.000000e+00 | NA | 2.031364e+109 | 0.9932 |
| *Frankel Neuro Classification before surgery: A* | -15.5265 | 0.000000e+00 | NA | 7.306899e+34 | 0.9901 |
| *Mobility level before surgery: independent* | -0.0752 | 9.275000e-01 | 0.3232 | 2.661500e+00 | 0.8870 |
| *Mobility level before surgery: assistance* | 0.9912 | 2.694400e+00 | 0.9271 | 7.852000e+00 | 0.0648 |
| *Mobility level before surgery: immobile* | -16.6752 | 0.000000e+00 | NA | 4.361696e+28 | 0.9894 |
| *Ataxia before surgery: yes* | -0.2790 | 7.566000e-01 | 0.3292 | 1.511500e+00 | 0.4628 |
| *Ataxia post-surgery: yes* | -0.8149 | 4.427000e-01 | 0.1234 | 1.034500e+00 | 0.1126 |
| *Neurological improvement after surgery* | 0.1658 | 1.180300e+00 | 0.4113 | 3.388600e+00 | 0.7541 |
| *Surgical type: dorsal* | 1.1022 | 3.010800e+00 | 0.7922 | 1.973980e+01 | 0.1572 |
| *Surgical type: ventral* | -16.5680 | 0.000000e+00 | NA | 3.136624e+39 | 0.9922 |
| *Surgical type: ventrodorsal/360°* | -0.4827 | 6.171000e-01 | 0.0931 | 2.400500e+00 | 0.5401 |
| *Surgical method: spondylodesis* | 16.5765 | 1.581522e+07 | 0.0000 | 1.594673e+168 | 0.9919 |
| *Surgical method: laminectomy* | 1.4391 | 4.216900e+00 | 1.1167 | 2.756750e+01 | 0.0640 |
| *Surgical method: kypho- or vertebroplasty* | -14.4711 | 0.000000e+00 | NA | 1.288780e+72 | 0.9917 |
| *Surgical method: corpectomy* | -0.1304 | 8.778000e-01 | 0.2841 | 2.508000e+00 | 0.8112 |
| *Staged surgery: yes* | 0.0924 | 1.096800e+00 | 0.2905 | 3.408700e+00 | 0.8802 |
| *Only stabilisation* | -1.5027 | 2.225000e-01 | 0.0341 | 8.396000e-01 | 0.0530 |
| *Postoperative Karnofsky Performance Score in percentage* | 0.0091 | 1.009100e+00 | 0.9795 | 1.043400e+00 | 0.5692 |
| *Pain postoperative: better* | 0.0498 | 1.051100e+00 | 0.3580 | 3.507700e+00 | 0.9303 |
| *Pain postoperative: worse* | -14.4711 | 0.000000e+00 | NA | 1.288780e+72 | 0.9917 |
| *Pain postoperative: same* | 0.1278 | 1.136400e+00 | 0.3394 | 3.352500e+00 | 0.8233 |
| *Response to OP pain: partial* | 0.1291 | 1.137900e+00 | 0.3855 | 3.237500e+00 | 0.8089 |
| *Response to OP pain: complete* | -0.1074 | 8.981000e-01 | 0.2391 | 2.770100e+00 | 0.8601 |
| *Response to OP pain: worse* | -14.4711 | 0.000000e+00 | NA | 1.288780e+72 | 0.9917 |
| *Repeat spinal surgery same Location expanding due to tumor progression* | 0.6771 | 1.968300e+00 | 0.2818 | 8.646600e+00 | 0.4152 |
| *Other surgery related complications: implant misplacement* | 0.5345 | 1.706700e+00 | 0.0861 | 1.155620e+01 | 0.6359 |
| Outcome: Bleeding ~ |  |  |  |  |  |
| *Time between diagnosis and spinal metastases and surgery* | 0.0006 | 1.000600e+00 | 0.9992 | 1.001600e+00 | 0.2959 |
| *Female sex* | -0.5131 | 5.986000e-01 | 0.1284 | 2.114000e+00 | 0.4572 |
| *Type of tumor: Prostate* | 0.9607 | 2.613600e+00 | 0.6524 | 9.089000e+00 | 0.1425 |
| *Type of tumor: Lung* | -1.3283 | 2.649000e-01 | 0.0142 | 1.436900e+00 | 0.2113 |
| *Type of tumor: Breast* | 0.2168 | 1.242100e+00 | 0.1818 | 5.192800e+00 | 0.7897 |
| *Type of tumor: Kidney* | -16.2307 | 0.000000e+00 | NA | 2.850569e+51 | 0.9928 |
| *Type of tumor: Liver* | 1.4740 | 4.366700e+00 | 0.5889 | 2.205170e+01 | 0.0939 |
| *Type of tumor: Colorectal* | 0.6855 | 1.984800e+00 | 0.1004 | 1.315560e+01 | 0.5422 |
| *Type of tumor: Esophagus* | -15.1758 | 0.000000e+00 | NA | 2.320745e+60 | 0.9925 |
| *Type of tumor: Urothelial* | 1.8142 | 6.136400e+00 | 0.2727 | 6.919710e+01 | 0.1513 |
| *Type of tumor: Thyroid* | -15.1758 | 0.000000e+00 | NA | 2.320745e+60 | 0.9925 |
| *Type of tumor: Other* | -16.2719 | 0.000000e+00 | NA | 4.237173e+42 | 0.9916 |
| *Synchronous bone metastases* | -0.5322 | 5.873000e-01 | 0.1510 | 1.957000e+00 | 0.4026 |
| *Metachronous bone metastases* | 0.5322 | 1.702700e+00 | 0.5110 | 6.624400e+00 | 0.4026 |
| *Visceral distant metastases* | -0.0438 | 9.571000e-01 | 0.2862 | 3.200900e+00 | 0.9420 |
| *Lung metastases* | -0.4164 | 6.594000e-01 | 0.1413 | 2.332400e+00 | 0.5466 |
| *Liver metastases* | 0.4547 | 1.575800e+00 | 0.4000 | 5.347300e+00 | 0.4802 |
| *Brain metastases* | -16.2470 | 0.000000e+00 | NA | 2.496759e+47 | 0.9923 |
| *Adrenal metastases* | -16.2470 | 0.000000e+00 | NA | 2.496759e+47 | 0.9923 |
| *Distant lymph node metastases* | -0.1772 | 8.376000e-01 | 0.1789 | 2.978400e+00 | 0.7982 |
| *Soft Tissue Metastases* | -0.6519 | 5.211000e-01 | 0.0778 | 2.093200e+00 | 0.4138 |
| *Other Distant Metastases* | 0.0553 | 1.056800e+00 | 0.2703 | 3.546900e+00 | 0.9311 |
| *Number of vertebral body metastases at time of surgery: 2 to 3* | 0.4797 | 1.615600e+00 | 0.4555 | 5.354000e+00 | 0.4347 |
| *Number of vertebral body metastases at time of surgery: more than 3* | 0.4605 | 1.584900e+00 | 0.4729 | 5.314800e+00 | 0.4453 |
| *Cervical bone lesion in surgery* | -0.4164 | 6.594000e-01 | 0.1413 | 2.332400e+00 | 0.5466 |
| *Thoracic bone lesion in surgery* | 17.4260 | 3.698424e+07 | 0.0000 | NA | 0.9924 |
| *Lumbar bone lesion in surgery* | 0.1904 | 1.209700e+00 | 0.3616 | 4.047600e+00 | 0.7520 |
| *Sacral bone lesion in surgery* | 1.1567 | 3.179500e+00 | 0.6467 | 1.229740e+01 | 0.1119 |
| *Comorbidity ACE 27 Score before surgery* | -0.0826 | 9.208000e-01 | 0.4036 | 1.907500e+00 | 0.8329 |
| *Comorbidity ACE 27 Score before surgery: 1* | 0.0438 | 1.044800e+00 | 0.3124 | 3.494200e+00 | 0.9420 |
| *Comorbidity ACE 27 Score before surgery: 2* | 0.2793 | 1.322200e+00 | 0.1931 | 5.553500e+00 | 0.7318 |
| *Comorbidity ACE 27 Score before surgery: 3* | -15.1758 | 0.000000e+00 | NA | 2.320745e+60 | 0.9925 |
| *Comorbidity: Renal disease* | -15.1912 | 0.000000e+00 | NA | 2.985518e+44 | 0.9913 |
| *Comorbidity: Cardiovascular disease* | -0.1098 | 8.960000e-01 | 0.1324 | 3.672700e+00 | 0.8916 |
| *Comorbidity: High blood pressure* | -0.4388 | 6.448000e-01 | 0.1830 | 2.118000e+00 | 0.4720 |
| *Comorbidity: Peripheral arterial disease* | 1.6639 | 5.280000e+00 | 0.6965 | 2.822940e+01 | 0.0641 |
| *Comorbidity: Depression* | -15.1758 | 0.000000e+00 | NA | 2.320745e+60 | 0.9925 |
| *Comorbidity: Polyneuropathy/Chronic neuromuscular disorder* | -15.1758 | 0.000000e+00 | NA | 2.320745e+60 | 0.9925 |
| *Comorbidity: Rheumatism* | 18.0882 | 7.171094e+07 | 0.0000 | NA | 0.9901 |
| *Comorbidity: Prothrombotic/haematological* | -13.1383 | 0.000000e+00 | NA | 1.174240e+123 | 0.9928 |
| *Comorbidity: Diabetes* | 0.7901 | 2.203700e+00 | 0.4575 | 8.220000e+00 | 0.2678 |
| *Comorbidity: COPD* | 0.8755 | 2.400000e+00 | 0.1197 | 1.673810e+01 | 0.4423 |
| *Comorbidity: Liver* | 1.1062 | 3.022700e+00 | 0.1478 | 2.277750e+01 | 0.3408 |
| *Comorbidity: Epilepsy* | -13.1383 | 0.000000e+00 | NA | 1.174240e+123 | 0.9928 |
| *Comorbidity: Obesity* | -17.3970 | 0.000000e+00 | NA | 6.314402e+53 | 0.9927 |
| *Comorbidity: Stroke* | -15.1682 | 0.000000e+00 | NA | 8.664131e+72 | 0.9932 |
| *Comorbidity: Asthma* | -14.1531 | 0.000000e+00 | NA | 1.607633e+72 | 0.9918 |
| *Comorbidity: Bronchitis* | -14.1531 | 0.000000e+00 | NA | 1.607633e+72 | 0.9918 |
| *Comorbidity: Ulcerative colitis* | -13.1383 | 0.000000e+00 | NA | 1.174240e+123 | 0.9928 |
| *Comorbidity: Pulmonary embolism* | -14.1457 | 0.000000e+00 | NA | 2.560450e+109 | 0.9933 |
| *Comorbidity: Sleep apnoea* | -13.1383 | 0.000000e+00 | NA | 1.174240e+123 | 0.9928 |
| *Comorbidity: Hypothyroidism* | 2.5148 | 1.236360e+01 | 0.4679 | 3.274724e+02 | 0.0826 |
| *Comorbidity: Dementia* | 1.8142 | 6.136400e+00 | 0.2727 | 6.919710e+01 | 0.1513 |
| *Comorbidity: Parkinson’s disease* | -13.1383 | 0.000000e+00 | NA | 1.174240e+123 | 0.9928 |
| *Comorbidity: Psoriasis* | 18.0882 | 7.171094e+07 | 0.0000 | NA | 0.9901 |
| *Smoker currently* | -1.4036 | 2.457000e-01 | 0.0132 | 1.330000e+00 | 0.1863 |
| *Smoker previously* | -0.2946 | 7.448000e-01 | 0.1105 | 3.027600e+00 | 0.7135 |
| *Height in cm* | -0.0046 | 9.954000e-01 | 0.9254 | 1.066000e+00 | 0.8982 |
| *Weight in kg* | -0.0295 | 9.710000e-01 | 0.9312 | 1.007200e+00 | 0.1383 |
| *BMI* | -0.1167 | 8.899000e-01 | 0.7719 | 1.010500e+00 | 0.0868 |
| *Instability before surgery: yes* | -17.3875 | 0.000000e+00 | NA | 6.212966e+54 | 0.9928 |
| *Instability before surgery: potential* | 0.2824 | 1.326300e+00 | 0.3739 | 6.201500e+00 | 0.6832 |
| *Instability before surgery: no* | 1.4430 | 4.233300e+00 | 0.8428 | 1.703920e+01 | 0.0522 |
| *Neurological symptoms before surgery: yes* | 1.4191 | 4.133300e+00 | 1.0407 | 2.754450e+01 | 0.0737 |
| *Frankel Neuro Classification before surgery* | 0.3015 | 1.351900e+00 | 0.8466 | 2.057400e+00 | 0.1731 |
| *Frankel Neuro Classification before surgery: E* | -1.3896 | 2.492000e-01 | 0.0374 | 9.899000e-01 | 0.0799 |
| *Frankel Neuro Classification before surgery: D* | 0.6822 | 1.978300e+00 | 0.5887 | 6.655100e+00 | 0.2595 |
| *Frankel Neuro Classification before surgery: C* | 0.2168 | 1.242100e+00 | 0.1818 | 5.192800e+00 | 0.7897 |
| *Frankel Neuro Classification before surgery: B* | 2.5148 | 1.236360e+01 | 0.4679 | 3.274724e+02 | 0.0826 |
| *Frankel Neuro Classification before surgery: A* | 0.2569 | 1.292900e+00 | 0.0670 | 7.884600e+00 | 0.8153 |
| *Mobility level before surgery: independent* | -1.2595 | 2.838000e-01 | 0.0610 | 9.977000e-01 | 0.0673 |
| *Mobility level before surgery: immobile* | -0.1098 | 8.960000e-01 | 0.1324 | 3.672700e+00 | 0.8916 |
| *Ataxia before surgery: yes* | 0.3115 | 1.365500e+00 | 0.6350 | 2.780900e+00 | 0.3984 |
| *Ataxia post-surgery: yes* | 0.4059 | 1.500700e+00 | 0.7265 | 2.940600e+00 | 0.2445 |
| *Surgical type: dorsal* | 1.5471 | 4.697900e+00 | 0.8707 | 8.726820e+01 | 0.1448 |
| *Surgical type: ventral* | -0.2248 | 7.987000e-01 | 0.0421 | 4.597000e+00 | 0.8354 |
| *Surgical type: ventrodorsal/360°* | -17.3505 | 0.000000e+00 | NA | 1.985397e+59 | 0.9933 |
| *Surgical method: spondylodesis* | 0.3019 | 1.352500e+00 | 0.2366 | 2.558360e+01 | 0.7798 |
| *Surgical method: laminectomy* | 1.0556 | 2.873600e+00 | 0.7210 | 1.918000e+01 | 0.1841 |
| *Surgical method: kypho- or vertebroplasty* | -14.1531 | 0.000000e+00 | NA | 1.607633e+72 | 0.9918 |
| *Surgical method: corpectomy* | -0.7596 | 4.678000e-01 | 0.1005 | 1.647200e+00 | 0.2701 |
| *Staged surgery: yes* | -1.2895 | 2.754000e-01 | 0.0148 | 1.495200e+00 | 0.2251 |
| *Only stabilisation* | -1.1180 | 3.269000e-01 | 0.0490 | 1.301800e+00 | 0.1593 |
| *Pain post OP: better* | -0.8162 | 4.421000e-01 | 0.1311 | 1.489100e+00 | 0.1783 |
| *Pain post OP: worse* | -14.1531 | 0.000000e+00 | NA | 1.607633e+72 | 0.9918 |
| *Pain post OP: same* | 0.9943 | 2.702700e+00 | 0.7994 | 9.155500e+00 | 0.1023 |
| *Response to OP pain: partial* | -0.7896 | 4.540000e-01 | 0.0975 | 1.598100e+00 | 0.2516 |
| *Response to OP pain: complete* | -0.1044 | 9.009000e-01 | 0.1921 | 3.209900e+00 | 0.8804 |
| *Response to OP pain: worse* | -14.1531 | 0.000000e+00 | NA | 1.607633e+72 | 0.9918 |
| *Repeat spinal surgery same Location expanding due to tumor progression* | 0.1437 | 1.154500e+00 | 0.0601 | 6.926400e+00 | 0.8956 |
| *Implant failure* | 0.3825 | 1.465900e+00 | 0.0755 | 9.125700e+00 | 0.7295 |
| *Other surgery related complications: implant misplacement* | 0.8755 | 2.400000e+00 | 0.1197 | 1.673810e+01 | 0.4423 |
| *Adjacent level metastasis instrumentation* | 1.1150 | 3.049500e+00 | 0.8451 | 1.033190e+01 | 0.0743 |

**Supplemental Table S5: Insignificant results of univariate regression models for implant failure, post-operative wound infections and post-operative bleeding/hematoma (p > 0.05).**

Supplemental Table S5 is provided for transparency to report clinically plausible variables evaluated in prespecified univariate analyses that did not demonstrate statistically significant associations (p<0.05) with implant failure, wound infection, or postoperative bleeding. Variables evaluated but not significantly associated with any of the three outcomes included demographic factors (age at diagnosis and surgery, sex, body mass index, height, weight), tumor characteristics (primary tumor type), metastatic burden and distribution (bone and visceral metastases, including pulmonary, hepatic, cerebral, adrenal, lymph node, soft tissue, and other sites), number of affected vertebral bodies, and anatomical spinal involvement (cervical, thoracic, lumbar, sacral). Additional non-significant variables comprised comorbidity burden (ACE-27 total score and individual comorbidities), preoperative clinical and neurological status (Karnofsky Performance Score, Frankel grade, neurological symptoms, mobility level, presence of ataxia), spinal instability, surgical approach and technique (dorsal, ventral, combined approaches; spondylodesis, laminectomy, kypho-/vertebroplasty, corpectomy), staged procedures, stabilization-only surgery, postoperative neurological improvement, postoperative pain response, and surgery-related complications such as implant misplacement or revision surgery.
